# Analysis for Joint Delay-Power Tradeoff with Buffer/Channel-Aware and Its FPGA Implementation in Wireless Sensor Networks

**DOI:** 10.3390/s20113114

**Published:** 2020-05-31

**Authors:** Joumana Dakkak, Saleh Eisa, Hesham M. El-Badawy, Ahmed Elbakly

**Affiliations:** 1 Department of Basic &Applied Sciences, Arab Academy for Science, Technology and Maritime Transport, Cairo 2033, Egypt; aelbakly1964@aast.edu; 2 Department of Electronics & Communications Engineering, Arab Academy for Science, Technology and Maritime Transport, Cairo 11799, Egypt; 3 Network Planning Department, National Telecommunication Institute (NTI), Cairo 11768, Egypt

**Keywords:** Internet of Things, Markov chain, delay-power tradeoff

## Abstract

In this paper, we aim to investigate the delay-power tradeoff problem which is attracting widespread interest due to its importance in wireless technology. This research has two main objectives. First, to assess the effect of different system parameters on the performance metrics. Second, to provide a solution for this optimization problem. A two-state, slow-fading channel is categorized into good and bad channel states. An adaptive transmission and random data arrivals are considered in our model. Each channel category has its own Markov chain, which is used in modeling the system. A joint Buffer-Aware and Channel-Aware (BACA) problem was introduced. In addition, an enhanced iterative algorithm was introduced for obtaining a sub-optimal delay-power tradeoff. The results show that the tradeoff curve is piecewise linear, convex and decreasing. Furthermore, a channel-aware system was investigated to provide analysis of the effect of system parameters on the delay and power. The obtained results show that the dominant factors that control the system performance are based on the arrival rate and the channel goodness factor. Moreover, a simplified field programable gate array (FPGA) hardware implementation for the channel aware system scheduler is presented. The implementation results show that the consumed power for the proposed scheduler is 98.5 mW and the maximum processing clock speed is 190 MHz.

## 1. Introduction

The Wireless Sensor Network (WSN) is constantly used for the development of applications like surveillance, area monitoring, health care monitoring, habitat monitoring, environmental sensing, and transportation sensing. With the Internet of Things (IoT) revolution, both business models and technologies are moving to a more advanced phase of innovation. IoT systems are usually operated by batteries. It will be very advantageous to extend battery life by reducing energy consumption, as well as providing self-powered devices or alternative energy sources to power the IoT systems uninterruptedly. Furthermore, for different IoT applications, varying from reliable and delay-sensitive applications to delay-tolerant applications, the network should be designed to meet different quality specifications. Nevertheless, faster data transfer typically requires devices to spend more energy. Therefore, a major challenge in this area is the trade-off between delay and power [1]. 

The first studies of energy-efficient scheduling under delay constraints problem were considered in [2]. A time-varying channel was considered and the problem was solved using Dynamic Programming (DP). In the same vein, in [3] a similar situation was considered as that in [2] but in the information-theoretic context. However, the results in [3] were enhanced in [4] and proved the presence of an optimal stationary average policy. In addition, it provided lower and upper limits for an optimum rate allocation policy, not obtained by [3]. Detailed examination of the types of power/rate control policies was discussed in [5]. The DP approach was used to achieve the optimal solution. All combinations of fixed/variable power with fixed/variable rate were analyzed. A similar problem was modeled in [6] using a basic cross-layer approach to obtain a closed-form solution using a fixed-modulation scheme. Only a single packet can be sent throughout the channel coherent time. In realistic cases, there is a strong probability that even more than a single packet will be sent throughout a channel coherent time. Power consumption, packet loss rate, and average delay of random scheduling were obtained by formulating Markov models.

Recent studies have been concerned with more realistic scenarios. The work discussed in [7] expanded the approach to a more general and practical scenario. Slow fading was the main focus of this work, where the channel time length allows many packets to be transmitted. The power consumption and average delay were evaluated on the basis of the Markov chain model. Linear programming (LP) was then designed to reduce the delay due to a fixed power limitation. Nevertheless, the same work considered the case of a short channel coherent time in [8]; where it would take many timeslots to send the packets thoroughly. 

In contrast, in [9] a new line of research was developed without considering channel variation. A different approach to the study of the adaptive transmission system has been suggested. Moreover, given an average power limit, the average delay was brought to a minimum. The work presented in [10] continued this analysis strategy, analyzed the issue through the Constrained Markov Decision Process (CMDP) framework, and presented analysis and results. While in [11], the authors generalized the Bernoulli arrival process to an arbitrary independent and identically distributed (i.i.d) distribution. They showed that the optimal curve representing power/delay tradeoff is piecewise linear, convex and decreasing with a threshold-based optimal policy. Furthermore, they developed their own algorithm to achieve the optimal delay-power tradeoff curve with the optimal policies. 

A broader perspective has been adopted in [12,13,14]. The work in [12] was extended to include the arbitrarily bursty data arrival considering two-state channel and fixed case transmission whereas multiple channel states with arbitrarily bursty data arrival were considered in [13]. Then in [14], complicated and more general wireless communication systems were adopted. The work employed an adaptive transmission to send bursty data traffics over multi-state fading channel and LP was used to solve the problem.

Additionally, the channel state needs to be updated in time due to the dynamically changing topology of the wireless network. In [15], the authors suggested two fully distributed protocols for time synchronization to enhance communication efficiency. Similarly, in [16] the paper developed a paradigm to analyze the performance of cooperative network synchronization. Together these studies provided important insights into the importance of wireless network time synchronization.

Furthermore, in [17] the authors suggested a network of wireless sensors which is operated by solar energy. An extensive performance analysis of the solar energy network system was presented. A broader perspective has been adopted by authors in [18] where the energy transfers, power allocations and optimum data rates were explored in order to minimize the overall delay in the energy harvesting of WSNs with interference channels in a time slot. In [19], the authors claim that routing has a significant importance in IoT applications. They suggested the Energy and Delay Aware Data aggregation for IoT in the routing protocol.

**Figure 1:**
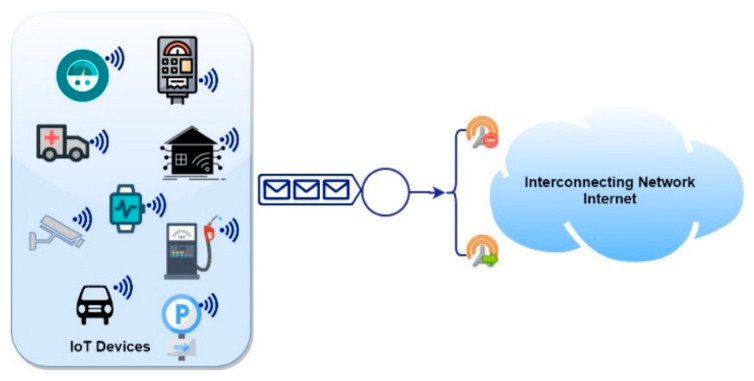
System under investigation.

Figure 1 shows an example of a typical communication scenario for different IoT applications. The presented work studies the interconnection backhauling structure between the core network element and the different IoT applications. We examine the performance and behavior of such a network. The collected data from different IoT applications are stored in a queue, which may cause undesirable delay. Since the power constraint is one of the major concerns of sensor networks, the scheduler of the system will decide, based on different system parameters and conditions, whether it will transmit the packets or not and how many packets should be transmitted. Therefore, these sensor networks will be devoted to minimize the delay under given power constraint. Accordingly, the current paper will investigate the joint optimization between the delay and the power consumption.

The concurrent work focuses on the case of wireless transmission. The objective of the paper is to analyze the relation between delay and power. We take different scenarios for wireless transmission under channel-aware system and BACA system. Various parameters such as the arrival packets distribution, the buffer capacity, and the number of maximum transmitted packets are taken into consideration. We start with the case of channel aware system. We obtain the delay-power tradeoff curve to provide a preliminary behavior for the delay and power tradeoff curve. Moreover, we propose the use of interpolation to find the relation between channel goodness factor and the average arrival rate with delay and power respectively. The use of interpolation provides an estimation for power and delay function, which can help operators to be aware of the power needed and the expected delay. An enhanced iterative algorithm is developed to deduce the delay-power suboptimal tradeoff curve and its corresponding policies under different channel conditions. Furthermore, hardware implementation based on FPGA for a channel-aware system was provided. The main objective of the hardware implementation is to promote a proof of concept for the channel aware scheduler realization in real life based on FPGA systems. 

This paper has been divided into nine sections, including this introductory section and the conclusion section. In Section 2, the system model and the parameters used are presented. The methodology used in the research and a description of the transmission probabilistic selection policy for the perfect and realistic channel state is introduced in Section 3. In addition, the Markov chain in Section 4 is formulated to help in solving the problem. Section 5 is concerned with evaluating the performance metrics, delay and power. An enhanced-iterative algorithm to obtain the sub-optimal policy under BACA system is presented in Section 6. In Section 7, numerical results and analysis are provided for the case of perfect channel state and realistic channel state. Channel-aware system is first introduced, then a BACA system enhanced iterative algorithm is discussed. FPGA hardware implementation for a channel aware scheduler is introduced in Section 8. 

## 2. System Model

**Figure 2:**
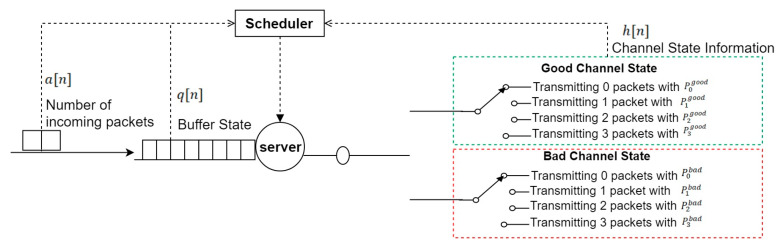
System model.

We study the case of transmitting bursty arrival packets through wireless links for IoT applications such as intelligent transportation system, smart agriculture, and smart cities. The proposed system model consists of a buffer, scheduler and a server as shown in Figure 2. The buffer is used to store the arrived packets with finite capacity. The information about the queue state q[n], state of channel h[n], and the arrived packets a[n] is collected by the scheduler to decide whether the server will transmit packets or not. The case of a slotted-time system is considered in which time is divided into slots for every transmission. Throughout the presented work, it is assumed that the channel is a two-state block-fading channel with probability (β). The system parameters and definitions are illustrated in the following subsections. 

First, packets are assumed to arrive in the system at the end of the n^th^ time slot with a maximum of A packets. The arrived packets are supposed to be independent and identically distributed (i.i.d) discrete random variables with a probability distribution given by

(1) $$Pr\left\{a\left(n\right)=a\right\}=\alpha_{a},a\in \left\{0,1,\ldots ,A\right\}$$

where $\alpha_{a}$ belongs to the interval [0,1] and

(2) $$\alpha_{a}\geq 0,\;\;\sum_{a=0}^{A}\alpha_{a}=1$$

The average arrival rate could be calculated by

(3) $$\bar{a}=\sum_{a=0}^{A}a\alpha_{a}$$

Then, s[n] represents the transmitted packet out of the system and completely served by the system in each timeslot n. The served packets are supposed to have a finite maximum value of S and s[n]∈{0,1,…, S}. To ensure the system stability, the packet flow out of the queue is assumed to be larger than or equal to the number of incoming packets, so S ≥ A.

For a finite buffer with capacity Q, let the length of the queue be dented by q[n] at the beginning of timeslot *n*, where q[n] ∈ {0, 1, …, Q}. A primary concern for the stored packets is the buffer overflow and underflow. Therefore, the number of packets served cannot surpass the number of packets in the queue. To avoid buffer underflow in our calculations:

(4) q[n]−s[n]≥ 0

And to avoid buffer overflow:

(5) q[n]−s[n]≤Q−A

The new queue state could be presented as:

(6) q[n+1]=q[n]−s[n]+a[n]

Time-varying slow-fading channel conditions are presumed in the system model. The fading process is approximately constant during a timeslot. It is assumed that the states of the channel are (i.i.d). First, the case of a good channel state is studied where the channel conditions are assumed to be perfect. Then, a bad channel state is introduced. Let h[n] indicate the channel state in the nth timeslot where h[n]=w and w∈{good, bad}.

The probability to have a good channel state is β, then (1−β) will be the probability to have a bad channel state

(7) $$\mathrm{Pr}\{\;h\left[n\right]=w=\{\begin{array}{c} ‘good’\}=\;\;\;\beta \\ \;\;\;‘bad\;’\;\}=1-\beta \end{array}$$

where β belongs to the interval 0<β≤1.

The power of transmitting s packets in w channel state is equal to $P_{s}^{w}$. Transmitting s packets in good channel state will cost power $P_{s}^{good}$ and transmitting s packets in bad channel state will cost power $P_{s}^{bad}$. However, transmitting packets under bad channel state will cost more power to overcome the difficulties in the bad channel state thus:

(8) $$P_{s}^{good}<P_{s}^{bad}$$

Additionally, transmitting more packets under the same channel state will cost more power:

(9) $$P_{1}^{w}<P_{2}^{w}$$

The system works in the following sequence: Towards the end of the n^th^ timeslot, information about the data arrival is collected by the system. The system will schedule the transmitted packets based on different parameters: buffer status, channel status and the information about the data arrival. In our problem, the goal is to minimize the delay given a power constraint.

Table 1 provides a full illustration of the parameters used in the system and its corresponding values.

## 3. Transmission Probabilistic Selection Policy

In this section, we try to investigate the transmission policy for realistic channel state. Taking the bad channel state into consideration, which has higher channel gain; more power will be needed to overcome the channel imperfections. To achieve low latency, the maximum allowed packets should be served which will cause high power consumption. Therefore, it is preferred to wait for a good channel state to have lower power consumption, but this causes a higher delay.

On the contrary, to consume lower power, fewer packets should be served, which will cause higher latency. The scheduler, based on the collected information, should compromise between these two important parameters and choose the suitable scheduling policy for each channel state.

Let $f_{q,s}^{w}$ denote the probability of transmitting s packets under w state with queue length equal to q

(10) $$f_{q,s}^{w}=Pr\left\{s\left(n\right)=s|q\left(n\right)=q,h\left(n\right)=w\right\}$$

thus,

(11) $$\sum_{s=0}^{S}f_{q,s}^{w}=1\forall q\in \left\{0,1,\ldots ,Q\right\},w\in \left\{1,2\right\}$$

As mentioned in Section 2, It has been noted that it is not possible to send a number of packets greater than that stored in the queue, so in the case of $s<q\;then\;f_{0,s}^{w}=0$ *(e.g.,* $f_{0,0}^{w}=1$*,* $f_{0,s}^{w}=0\;\forall \;s\neq 0$). This condition helps the system avoid buffer underflow.

Also, to avoid overflow, $f_{q,s}^{w}=0$ *if* s<q−Q+A. For example, in case of maximum queue state q=Q has occurred, all arrived packets should be served (s=A) to avoid packets drop ($f_{Q,S}^{w}=1$ and $f_{Q,s}^{w}=0\forall \;s\neq S$).

To summarize, we find that:

(12) $$f_{q,s}^{w}=0,\;\forall \;s>q\;\;or\;s<q-Q+A$$

In our case, two transmission policies are proposed, one for the good channel state and one for the bad channel state $F^{good}$, $F^{bad}$ respectively.

(13) $$F^{good}=\;\;\;\;\left[\begin{array}{cc} \begin{array}{cc} 1 & 0\\ f_{1,0}^{good} & f_{1,1}^{good} \end{array} & \begin{array}{cc} 0 & 0\\ 0 & 0 \end{array}\\ \begin{array}{cc} f_{2,0}^{good} & f_{2,1}^{good}\\ 0 & f_{3,1}^{good}\\ \begin{array}{c} 0\\ 0 \end{array} & \begin{array}{c} 0\\ 0 \end{array} \end{array} & \begin{array}{cc} f_{2,2}^{good} & 0\\ f_{3,2}^{good} & f_{3,3}^{good}\\ \begin{array}{c} f_{4,2}^{good}\\ 0 \end{array} & \begin{array}{c} f_{4,3}^{good}\\ 1 \end{array} \end{array} \end{array}\right],F^{bad}=\;\;\left[\begin{array}{cc} \begin{array}{cc} 1 & 0\\ f_{1,0}^{bad} & f_{1,1}^{bad} \end{array} & \begin{array}{cc} 0 & 0\\ 0 & 0 \end{array}\\ \begin{array}{cc} f_{2,0}^{bad} & f_{2,1}^{bad}\\ 0 & f_{3,1}^{bad}\\ \begin{array}{c} 0\\ 0 \end{array} & \begin{array}{c} 0\\ 0 \end{array} \end{array} & \begin{array}{cc} f_{2,2}^{bad} & 0\\ f_{3,2}^{bad} & f_{3,3}^{bad}\\ \begin{array}{c} f_{4,2}^{bad}\\ 0 \end{array} & \begin{array}{c} f_{4,3}^{bad}\\ 1 \end{array} \end{array} \end{array}\right]$$

These two policies could be combined and the system transmission policy F is sized as (Q+1)×(S+1)×W dimensional probabilistic matrix F whose element in the ${\left(k+1\right)}^{th}$ row, ${\left(s+1\right)}^{th}$ column and $w^{th}$ page is $f_{q,s}^{w}$ as shown in equation (13) for the case of Q=5 and S=3.

To reach the optimality of power-delay tradeoff relation, under deterministic threshold-based policy F, and given an average power constraint $P^{th}$, we must bring the average delay to a minimum. Let $P^{F}$ and $D^{F}$ represent the average power consumption and the average queueing delay of policy F.

(14) $$\{\begin{array}{c} \underset{F}{\min\;}D^{F}\\ s.t\;P^{F}\leq P^{th} \end{array}$$

## 4. Markov Chain Formulation

**Figure 3:**
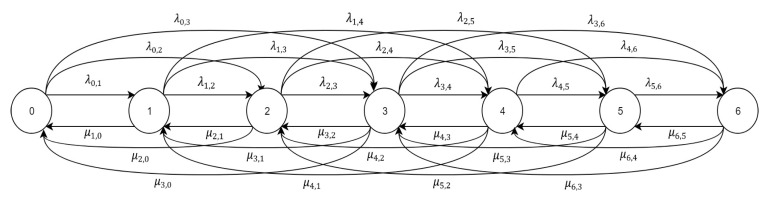
Markov chain for Q=6,A=3,S=3.

The scheduling problem was modeled using Markov chain as in Figure 3. Markov chain was chosen because it is one of the most feasible ways to describe our model as we assume that the next state depends only on the current state. The Markov chain is divided into Q+1 states, which represent the queue length states. The transition probability from state i to state j is denoted by $\tau_{i,j}$.

For j≥i, the transition probability will be denoted by $\lambda_{i,j}$ and for j<i it will be denoted by $\mu_{i,j}$. It is not possible to transit more than A steps forward and S steps backward. The transition probability is assumed to be the sum of two transition probabilities; one under the good channel state multiplied by β ratio and the other under the bad channel state multiplied by (1−β). The transition probability to transit from state i to state j under wth channel state for j≥i and for j<i will be denoted by $\lambda_{i,j}^{w}$,$\mu_{i,j}^{w}$ respectively. $\lambda_{i,i}$ for i=0, …,Q are removed to keep the diagram visible.

(15) $$\tau_{i,j}=\{\begin{array}{cc} \lambda_{i,j} & j\geq i\;\\ \mu_{i,j} & j<i \end{array}$$

**Figure 4:**
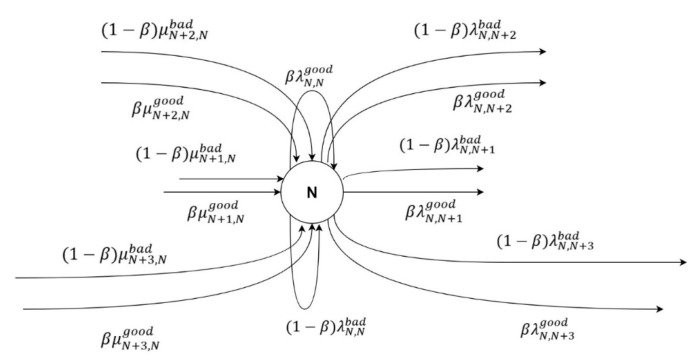
The Markov chain illustration for one state example.

The actual state is the combination of the good state with ratio β and with the bad channel state with ratio 1−β as shown for state N in Figure 4.

The state transition probability $\tau_{i,j}^{w}$ can be calculated as

(16) $$\tau_{i,j}^{w}=\sum_{s=\;\max\left\{0,\;i+A-Q,i-j\right\}}^{s=\min\left\{S,i,\;\;A-j+i\right\}}\alpha_{j-i+s}f_{i,s}^{w}$$

And the total $\lambda_{i,j}$ could be derived as

(17) $$\lambda_{i,j}=\beta \tau_{i,j}^{good}+\left(1-\beta \right)\tau_{i,j}^{bad}\;\;\;\;\;j\geq i$$

And the total $\mu_{i,j}$ could be derived as

(18) $$\mu_{i,j}=\beta \tau_{i,j}^{good}+\left(1-\beta \right)\tau_{i,j}^{bad}\;\;\;\;\;j<i$$

The transition probability matrix is denoted as Λ with a matrix dimension (Q+1)×(Q+1) whose element in the (i)th column (j)th row is $\tau_{i,j}$.

The probability distribution under steady-state is defined as π. Let π(q) denote the steady-state probability of state (q) under stationary conditions. Therefore, the stationary distribution π=[π(0)*,* π(1)*,…,* π(Q)].

To obtain the steady-state probability, we can follow the following technique for solving the stationary equations

(19) $$\vec{\pi }.\Lambda =\vec{\pi },\;1^{T}.\pi =1$$

An equivalent way to express the equation is using eigenvalue *(*ϵ) and eigenvector $\left(\vec{v}\right)$ such that

(20) $$\left[\Lambda -\epsilon I\right]\vec{v}=0$$

In this case, the eigenvalue ϵ=1, for the eigenvector $\vec{v}=\vec{\pi }$. Thus,

(21) $$\left[\Lambda -I\right]\vec{\pi }=0\;and\;1^{T}.\vec{\pi }=1$$

Such that $1={\left[1,1,\ldots ,1\right]}^{T}$ and $0={\left[0,0,\ldots ,0\right]}^{T}$, by taking B=[Λ−I]

(22) $$[\begin{array}{c} 1^{T}\\ B\left(0\right)\\ B\left(1\right)\\ .\\ .\\ .\\ B\left(Q-1\right) \end{array}]\vec{\pi }=[\begin{array}{c} 1\\ 0\\ 0\\ .\\ .\\ .\\ 0\; \end{array}]$$

By solving Equation (22), steady-state probability can be achieved as in Equation (23)

(23) $$\vec{\pi }={\left[\begin{array}{c} 1^{T}\\ B\left(0\right)\\ B\left(1\right)\\ .\\ .\\ .\\ B\left(Q-1\right) \end{array}\right]}^{-1}\;[\begin{array}{c} 1\\ 0\\ 0\\ .\\ .\\ .\\ 0\; \end{array}]$$

## 5. Performance Metrics 

The performance of the proposed model will be evaluated in this section. Across the paper, the system performance may be evaluated by means of the contradicting relation between delay and power consumption for different arrival patterns with different channel goodness factors (wireless channel state.) 

### 5.1. System Delay

The system delay will be evaluated based on the derived state transition as stated in Section 3. Little’s law [20] will determine the estimated waiting time in the system. The mean queue length in the system could be calculated by $\sum_{q=0}^{Q}q\pi \left(q\right)$, which could denote the average packets stored in the system. The average arrival rate is calculated by Equation (3) in Section 2.

According to Little’s law, the expected waiting time which is the delay (D) in this case is given by:

(24) $$D=\frac{1}{\bar{a}}\overset{Q}{\underset{q=0}{\sum }}q\pi \left(q\right)$$

### 5.2. Estimated Power Consumption

The estimated power consumption will be determined based on the transition probability matrix as well as the channel goodness factor (β) and the policy selection criteria matrix F. 

For having q packets in the queue, let $P_{s}^{good}$ denote the power of transmitting s packets under good channel state with selection probability $f_{q,s}^{good}$ and let $P_{s}^{bad}$ denote transmission under bad channel condition with selection probability $f_{q,s}^{bad}$. The total power (P) is expressed as follows:

(25) $$P=\beta \sum_{q=0}^{Q}\pi \left(q\right)\sum_{s=0}^{S}P_{s}^{good}f_{q,s}^{good}+\left(1-\beta \right)\sum_{q=0}^{Q}\pi \left(q\right)\sum_{s=0}^{S}P_{s}^{bad}f_{q,s}^{bad}$$

## 6. BACA Selection Policy Enhanced Algorithm

We propose an enhanced iterative algorithm (Algorithm 1). The purpose of this algorithm is to achieve a sub-optimal piecewise linear tradeoff curve with less complexity than solving it with the available methods and algorithms. The proposed algorithm depends on both the channel and buffer state. It was prepared using a similar procedure as in the algorithm used in [11], but for wireless transmission which makes the algorithm more complex to solve as the policy matrix F will become a three-dimensional matrix instead of a two-dimensional matrix. 

The proposed iterative algorithm starts from the highest-power lowest-delay point. This point is achieved by transmitting as many packets as possible and applying the corresponding three-dimensional matrix F as in step 1 in Algorithm 1. We calculate the delay and power at that point and store them in $D_{old},P_{old}$ as in step 2 and 3. We start from this point as it is easy to determine and it will be considered our first point $\left(P_{old},D_{old}\right)$. We change the transmission policy in one threshold as in step 4 to obtain a new point. We calculate the delay and power for this new point and the slope between it and the first point. With each change we make in the transmission policy we get a new possible point (candidate). So, we obtain a number of candidate points each with a different slope. The points will be then tested one by one to eventually choose one of them to be the next point on the curve that represents the lower power and minimum slope as in steps 5–10. We draw the connecting line as in step 11. The same steps are repeated until we reach the tradeoff curve and achieve the lowest power consumption point. The obtained curve gives an optimized relation between the power and delay for the wireless communication system.
**Algorithm 1** Enhanced Iterative Algorithm 1:Construct three-dimensional matrix policy F with maximum transmission in good channel state and bad channel state  2:Calculate delay (D) and power (P)
 3:$F_{old}\leftarrow F$, $D_{old}\leftarrow D$, $P_{old}\leftarrow P$, slope←∞ 4:Construct new three dimensional $\bar{F}$ matrix different in one queue state threshold in good or bad channel state threshold  5:**if**F is Feasible and threshold-based 6:Calculate delay $\left(\bar{D}\right)$ and power $\left(\bar{P}\right)$ and slope between ($P_{old},D_{old})$ and calculated point $\bar{\to slope}$
 7:**If**$\bar{P}<P_{old}$ & $\bar{slope}<slope$
 8:$slope\leftarrow \bar{slope}$, $F_{new}\leftarrow \bar{F}$, $D_{new}\leftarrow \bar{D}$, $P_{new}\leftarrow \bar{P}\;$ 9:**Repeat:** from step 4 for all different thresholds 10:**Until:** all thresholds are considered  11:Draw line connecting ($P_{old},D_{old})$ and $\left(P_{new},D_{new}\right)$
 12:$F_{old}\leftarrow F_{new}$, $D_{old}\leftarrow D_{new}$, $P_{old}\leftarrow P_{new}$, slope←∞ 13:**Repeat:** from step 4 for all points  14:**Until:** the lowest power consumption point achieved 

## 7. Numerical Results and Analysis

The system performance metrics, delay and power, are obtained based on numerical analysis for both channel-aware system and for channel-aware buffer-aware system.

A practical scenario with adaptive transmission was considered. The proposed model is considering wireless transmission for realistic channel state, which takes into account the good and bad channel states. The operational parameters are chosen similar to that in [11], which studied the case of wired transmission for good channel state only. Whereas, the power under bad channel state is assumed to be four times that of the good channel state as in [6]. Results for the proposed model were verified by applying the enhanced algorithm to good channel state condition first (β=1), and the tradeoff results were verified. Then, the realistic channel state was considered and studied.

Moreover, the assumed bandwidth =1 MHz, the timeslot length =10 ms, and the target bit error rate $\mathrm{BER}=10^{-5}$. The assumed packet contains 10,000 bits. However, it is assumed that the maximum arrival packets A=3, which means that we could receive 0,1,2, or 3 packets, and the maximum served packets S=3, which means that we could transmit 0,1,2, or 3 packets. Assume the one-sided noise power spectral density $N_{0}=-150\;\mathrm{dBm}/\mathrm{Hz}$.

The transmitting power for various transmitting rates in a good channel state and a bad channel state are listed as seen in Table 2. For the rest of the analysis, we set the buffer size Q=10. 

We begin by investigating a channel-aware system. We are trying to achieve the delay-tradeoff curve and the related policies. To exactly determine how the system parameters affect the performance metrics, bicubic interpolation is used to correlate the relation. Following this, a BACA system is introduced. We try to deduce the delay-power tradeoff using an enhanced algorithm. We obtain a suboptimal piecewise linear tradeoff curve.

### 7.1. Channel-Aware Selection Policy 

A case study approach for channel aware system is used to provide a tentative behavior for the delay and power tradeoff curve. This approach depends on the channel state only. 

This study confirms the importance of channel goodness factor as it shows how changing the channel conditions will impact final results.

The system was simulated and evaluated under the assumption that under good channel state the system will transmit as much packets as possible (e.g., $S^{good}=3$). Under bad channel state, there are three basic cases currently being adopted:

- The first case considered: transmit 100% of allowed packets (e.g., $S^{bad}=3$)
- The second case considered: transmit 66.667% of allowed packets. (e.g., $S^{bad}=2$)
- The third case considered: transmit 33.33% of allowed packets. (e.g., $S^{bad}=1$)

**Figure 5:**
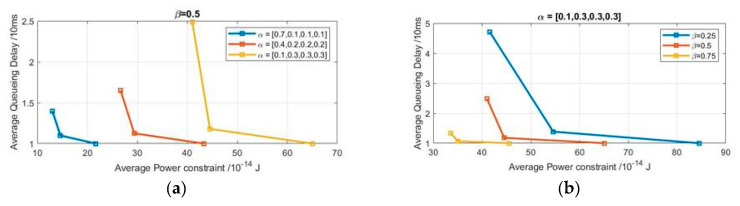
Realistic channel state condition channel-aware tradeoff curve for (**a**) β=0.5, (**b**) for α=[0.1,0.3,0.3,0.3].

In this section, we will investigate the effect of bursty arrival packets and their probability distribution on the channel-aware system. Then, we will study the effect of channel goodness factor, which is a key factor in the performance since it had a huge impact on the results. The channel goodness factor ranged from 0.01–1 as 0.01 describes a high probability of having a bad channel state and 1 describes a high probability of having a good channel state. The results obtained from the preliminary analysis of channel-aware delay-power tradeoff are presented in Figure 5.

Each point in Figure 5 represents one of the considered cases. The first point achieved is at the bottom of the curves and represents the first case, which has the lowest delay and the highest power, as we transmit three packets under good or bad channel state. The second and third points represent the second and the third cases respectively. 

Figure 5a provides the results obtained from the effect of changing the average arrival rate on the delay and power under same channel conditions (β=0.5). Figure 5b provides the results obtained from the effect of changing the channel goodness factor on the delay and power given the same arrival distribution α=[0.1,0.3,0.3,0.3].

As shown in Figure 5, the delay-power tradeoff curves are decreasing and convex. It shows that with successive increase in the average arrival rate under same channel conditions, having higher arrival rate, the system experiences higher latency and costs more power. Also, there has been a steep decrease in the performance metrics as the channel goodness factor increases. This finding confirms the importance of the channel goodness factor as an effective aspect. To provide a full vision of the effect of the parameters on the performance metrics, interpolation was considered as it provides an estimation for the performance metrics given different parameters. 

### 7.2. Bicubic Interpolation

Interpolation was applied to channel-aware system to provide a deeper insight into the relation between performance metrics and different system parameters. This helps to provide an estimation for power and delay function, which can help operators to be aware of the power needed, the expected delay, and also to sustain a feasible range of channel goodness factor. Bicubic produces noticeably improved prediction for the asymptotic power/delay relation. This relation has more accuracy than the interpolation techniques such as bi-linear method. So, choosing the bi-cubic interpolation may present the ideal combination of processing time and output accuracy.

To model any function for two parameters x and y

(26) $$T\left(x,y\right)=\sum_{i=0}^{3}\sum_{j=0}^{3}c_{ij}{\left(x\right)}^{i}y^{j}$$

We can rewrite the function as

(27) $$T\left(x,y\right)=\left[\begin{array}{ccc} {\left(x\right)}^{3} & {\left(x\right)}^{2} & \begin{array}{cc} {\left(x\right)}^{1} & {\left(x\right)}^{0} \end{array} \end{array}\right]\;\left[\begin{array}{cc} \begin{array}{cc} c_{3,3} & c_{3,2}\\ {\;c}_{2,3} & c_{2,2}\; \end{array} & \begin{array}{cc} c_{3,1} & c_{3,0}\\ {\;c}_{2,1} & c_{2,0}\; \end{array}\\ \begin{array}{cc} c_{1,3} & c_{1,2}\\ c_{0,3} & c_{0,2} \end{array} & \begin{array}{cc} c_{1,1} & c_{1,0}\\ c_{0,1} & c_{0,0} \end{array} \end{array}\right]\left[\begin{array}{c} y^{3}\\ y^{2}\\ \begin{array}{c} y^{1}\\ y^{0} \end{array} \end{array}\right]$$

Given 4 × 4 known data points, we have

$$X=\left[\begin{array}{ccc} {x_{0}}^{3} & \cdots & {x_{0}}^{0}\\ \vdots & \ddots & \vdots \\ {x_{3}}^{3} & \cdots & {x_{3}}^{0} \end{array}\right]\;Y=\left[\begin{array}{ccc} {y_{0}}^{3} & \cdots & {y_{3}}^{3}\\ \vdots & \ddots & \vdots \\ {y_{0}}^{0} & \cdots & {y_{3}}^{0} \end{array}\right]$$

Then

(28) T=XCY

To get the coefficients for the modeled function

(29) $$C=X^{-1}T{\left(Y\right)}^{-1}$$

For each performance metric, we provide a dataset for average arrival rate and beta function and we calculate the coefficients. 

First, bicubic interpolation is used to correlate the relation among the average arrival rate, the channel goodness factor and delay. We apply the given data set (same variance is considered)

$$\bar{a}=\left[0.6,\;1.2,\;1.8,\;2.4\right]\;\;\;\;\;\;\beta =\left[0.01,0.33,0.67,1\right]$$

(30) $$D\left(\bar{a},\beta \right)=\sum_{i=0}^{3}\sum_{j=0}^{3}c_{ij}{\left(\bar{a}\right)}^{i}\beta^{j}$$

Assume that

$$\bar{A}=\left[\begin{array}{ccc} {{\bar{a}}_{1}}^{3} & \cdots & {{\bar{a}}_{1}}^{0}\\ \vdots & \ddots & \vdots \\ {{\bar{a}}_{4}}^{3} & \cdots & {{\bar{a}}_{4}}^{0} \end{array}\right]\;{Β}^{T}=\left[\begin{array}{ccc} {\beta_{1}}^{3} & \cdots & {\beta_{1}}^{0}\\ \vdots & \ddots & \vdots \\ {\beta_{4}}^{3} & \cdots & {\beta_{4}}^{0} \end{array}\right]\;\;C=\left[\begin{array}{ccc} c_{33} & \cdots & c_{30}\\ \vdots & \ddots & \vdots \\ c_{03} & \cdots & c_{00} \end{array}\right]$$

We will find that

(31) $$D=\bar{A}\;C\;{Β}^{T}$$

To get C

(32) $$C={\left(\bar{A}\right)}^{-1}\;D\;{\left({Β}^{T}\right)}^{-1}$$

By applying the given data set

$$C=\left[\begin{array}{cc} \begin{array}{cc} 7.4701 & -11.9921\\ -28.2929 & 46.1872\; \end{array} & \begin{array}{cc} 2.9754\; & 1.5467\\ -12.8966 & -4.9977 \end{array}\\ \begin{array}{cc} 32.0830 & -52.8875\\ -10.6858 & 17.7395 \end{array} & \begin{array}{cc} 15.8675\; & 4.9369\\ -5.7705 & -0.2833 \end{array} \end{array}\right]$$

To get delay at any point

(33) $$D\left(\bar{a},\beta \right)=\left[\begin{array}{ccc} {\left(\bar{a}\right)}^{3} & {\left(\bar{a}\right)}^{2} & \begin{array}{cc} {\left(\bar{a}\right)}^{1} & {\left(\bar{a}\right)}^{0} \end{array} \end{array}\right]\;C\;{\left[\begin{array}{ccc} {\left(\beta \right)}^{3} & {\left(\beta \right)}^{2} & \begin{array}{cc} {\left(\beta \right)}^{1} & {\left(\beta \right)}^{0} \end{array} \end{array}\right]}^{T}$$

Network operator may use this design equation for setting a certain requirement under given power constraints. 

Following this, bicubic interpolation is used again to correlate the relation among power, average arrival rate and channel goodness factor.

(34) $$P\left(\bar{a},\beta \right)=\sum_{i=0}^{3}\sum_{j=0}^{3}k_{ij}{\left(\bar{a}\right)}^{i}\beta^{j}$$

Assume that

$$K=\left[\begin{array}{ccc} k_{33} & \cdots & k_{30}\\ \vdots & \ddots & \vdots \\ k_{03} & \cdots & k_{00} \end{array}\right]$$

We will find that

(35) $$P=\bar{A}\;K\;{Β}^{T}$$

To get K

(36) $$K={\left(\bar{A}\right)}^{-1}\;P\;{\left({Β}^{T}\right)}^{-1}$$

By applying the given data set

$$K=\left[\begin{array}{cc} \begin{array}{cc} 18.0431 & -35.5982\\ -66.0866 & 127.3214\; \end{array} & \begin{array}{cc} \;44.2723\; & -11.9487\\ -157.9242\; & 43.6894 \end{array}\\ \begin{array}{cc} 73.5752 & -139.6484\\ -24.5379 & 48.5640\; \end{array} & \begin{array}{cc} 145.6978\; & -12.4413\\ -55.8699 & 16.0939 \end{array} \end{array}\right]$$

To get power at any point

(37) $$P\left(\bar{a},\beta \right)=\left[\begin{array}{ccc} {\left({\bar{a}}_{1}\right)}^{3} & {\left({\bar{a}}_{1}\right)}^{2} & \begin{array}{cc} {\left({\bar{a}}_{1}\right)}^{1} & {\left({\bar{a}}_{1}\right)}^{0} \end{array} \end{array}\right]\;K\;{\left[\begin{array}{ccc} {\left(\beta_{1}\right)}^{3} & {\left(\beta_{1}\right)}^{2} & \begin{array}{cc} {\left(\beta_{1}\right)}^{1} & {\left(\beta_{1}\right)}^{0} \end{array} \end{array}\right]}^{T}$$

**Figure 6:**
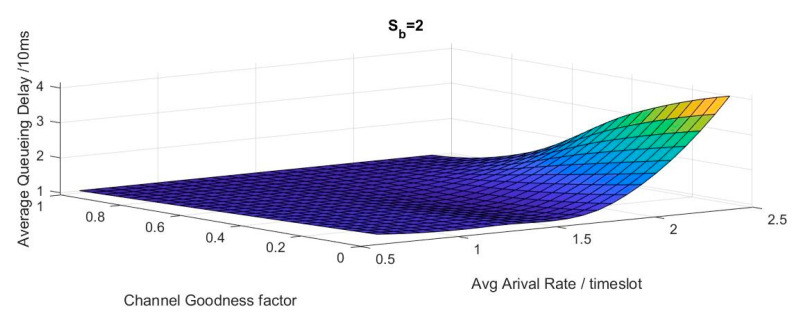
Realistic channel-aware system delay performance interpolation.

In Figure 6, the interpolation is used to correlate the system delay according to channel goodness factor β and average arrival rate $\bar{a}$. It shows that by increasing the average arrival rate in good channel condition, the system will not suffer from huge delay. Vice versa, by increasing the arrival rate and operating in a very bad channel condition β around 0.01, the system will behave badly and the delay will be at its highest point.

**Figure 7:**
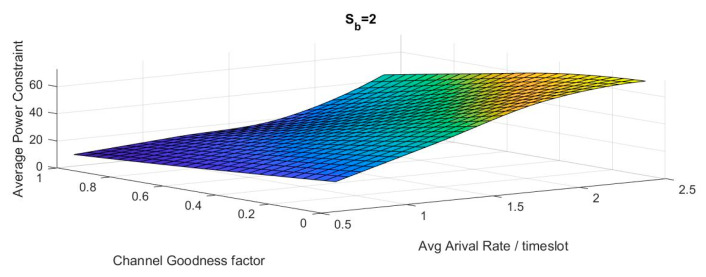
Realistic channel-aware system power performance interpolation.

Figure 7 represents the interpolation to correlate the system average power according to channel goodness factor and average arrival rate. It was found that the behavior of both delay and power are monotonically the same. The figure also shows that by increasing the average arrival rate in good channel condition, the system will not consume high power. By increasing the arrival rate and operating in a very bad channel condition β around 0.01, the system will behave badly and will consume huge power.

### 7.3. BACA Selection Policy Enhanced Algorithm

To validate the suggested algorithm, we used the enhanced algorithm proposed in section [6] with the perfect channel state with β=1 to achieve an optimum buffer-aware delay-power tradeoff curve and the related optimal policies.

**Figure 8:**
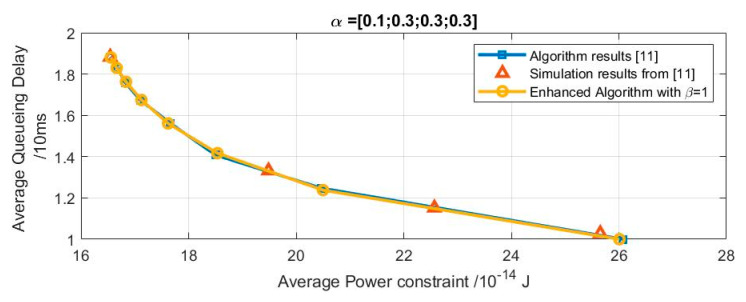
Perfect channel state tradeoff curve verification: results obtained from proposed model and results obtained from previous work.

As shown in Figure 8, the curves are consistent with curves obtained in [11].

**Figure 9:**
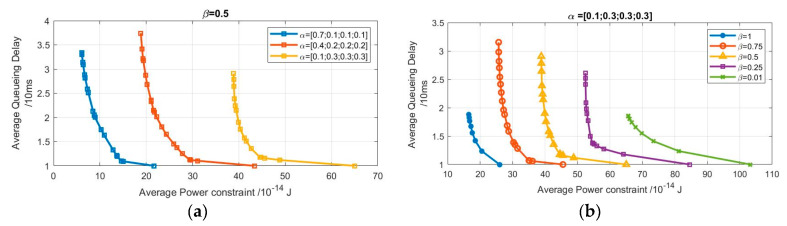
BACA system suboptimal tradeoff curves for: (**a**) β=0.5,(**b**) α=[0.1,0.3,0.3].

Following this, we applied the BACA iterative enhanced algorithm to a realistic channel state. We obtained the piecewise linear power-delay tradeoff curve for wireless transmission. It differs from the previous work depending on both the channel and buffer state. Changes in channel goodness factor and the burstiness of the arrivals are highlighted in Figure 9.

In the delay-power tradeoff curve, each point represents a policy that is different in one threshold than the previous one. The tradeoff curve is piecewise linear, convex and decreasing. These results match those observed in earlier studies on the power-delay tradeoff curve.

First, we studied the relationship between the tradeoff curve and the bursty incoming packets. To examine this relationship, the same channel goodness factor was used (β=0.5) as shown in Figure 9a. Following the increase of the average arrival rate, which is considered as a heavier workload for the system, a significant increase in power consumption has occurred.

Then, we investigated the relationship between the tradeoff curve and the channel goodness factor. To assess this relationship, the same arrival distribution was used (α=[0.1;0.3;0.3;0.3]) as shown in Figure 9b. It was found that with successive decrease in the channel goodness factor, the tradeoff curve moved further to a significant power increase.

Under certain power constraints, using the BACA algorithm provides the minimum delay and its corresponding policy. This may be useful for operators and network deployers in order to achieve more energy-efficient solutions with minimum delay.

## 8. Proposed Scheduler Hardware Implementation

**Figure 10:**
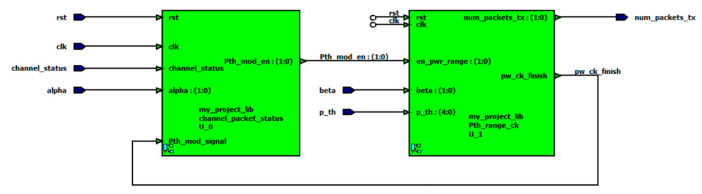
Block diagram of the proposed channel aware scheduler.

In this section, we propose an FPGA hardware implementation for a scheduler that is designed according to a channel aware policy algorithm. The proposed scheduler consists of two main blocks, the channel_packet_status and the Pth_range_ck, as shown in Figure 10. The channel_packet_status block is used to check the channel status (good/bad) and the average arrivals (alpha). The inputs for first block are the channel_ status, alpha and the Pth_mod_signal in addition to the clock and reset signals. The output of the first block is a 2 bit enable signal (Pth_mod_en) that is used to enable the second block. The second block is Pth_range_ck, which is used to check the range of the desired input power and suitable number of packets to transmit based on the proposed policy. The inputs of this block are the power threshold (p_th), the channel goodness factor (beta) and the enable signal (output from first block) in addition to the clock and reset signals.

### 8.1. Design of the Channel and Packet Status Block

**Figure 11:**
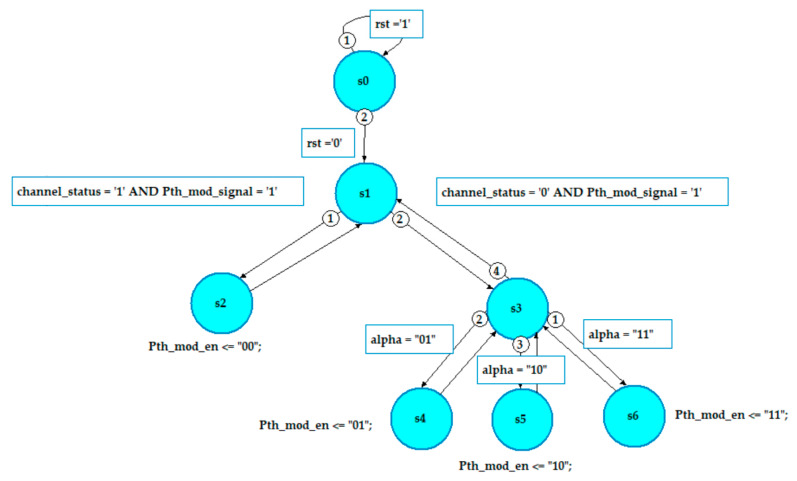
FSM for the proposed channel_packet_status block.

The channel_packet_status block is designed using finite state machines (FSMs). The block checks whether the channel status is good (represented by logic 1) or bad (represented by logic 0). If the channel status is good, the enable signal is set to “00” otherwise it is set to “01”, “10” or “11” according to the value of alpha is shown in Figure 11.

### 8.2. Design of the Power Threshold Check Block 

**Figure 12:**
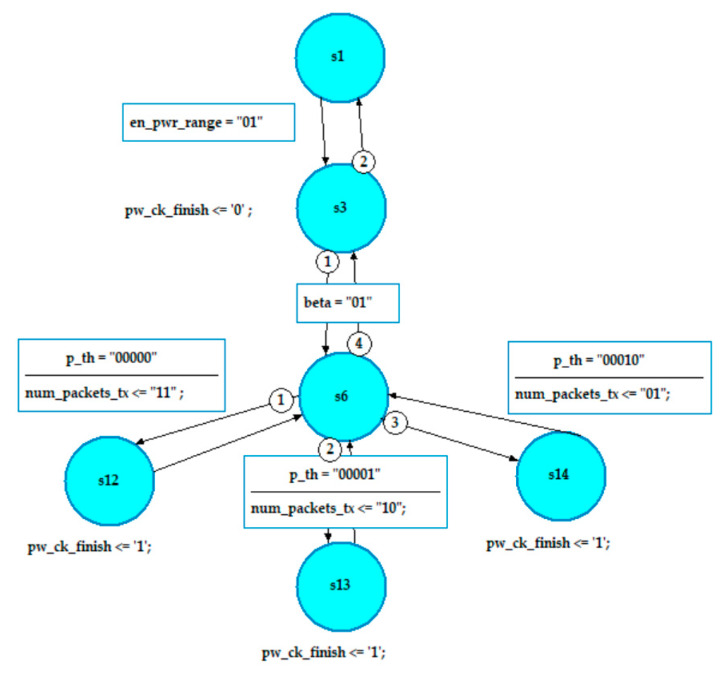
Part of the FSM for the proposed Pth_range_ck block.

Similar to the first block, the Pth_range_ck block is designed using finite state machines (FSMs) as shown in Figure 12. The design starts by enabling the power range according to the output signal of the first stage.

Then the probability of the channel goodness (beta) is checked. The goodness of the channel in bad state has three values, “01” which represents 75% channel goodness, and “10” and “11” that represent 50% and 25% channel goodness probabilities, respectively. The power threshold is represented by a 5-bit signals (p_th) to cover all the power range according to the range specified by the policy. The number of transmitted packets (num_packets_tx) is chosen according to the values of beta and the specified power threshold. The maximum number of transmitted packets is 3 and represented by “11”, and the minimum is 1 packet and represented by “01”. 

### 8.3. Simulation and Synthesis Results 

The proposed scheduler is implemented using Cyclone^®^ IV E FPGA from Intel (Altera). The targeted FPGA chip comprises of 114 k programmable logic elements (LEs), 388 embedded memory (Kbits), four PLLs, and 532 multipliers (9-bit) [21]. Table 3 shows the allocated resources for the implemented scheduler as well as the power consumption and maximum processing clock speed. The proposed scheduler is implemented using 143 LEs and 41 registers. The consumed power is 98.5 mW and the maximum processing clock speed is 190 MHz.

**Figure 13:**
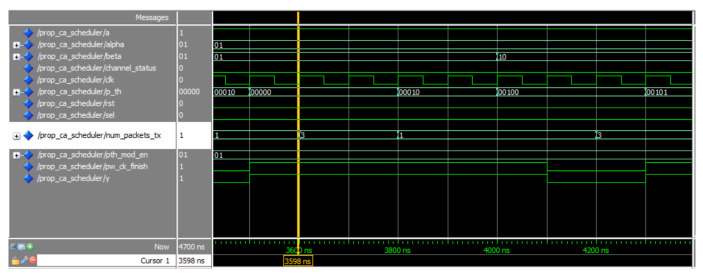
Simulation of the proposed channel aware scheduler.

Figure 13 presents the simulation results for the proposed channel aware scheduler. The number of transmitted packets changes according to the channel state, alpha, beta, and the power threshold. For example, at 3500 ns, the channel state is ‘0’, which represents bad channel state. The value for the packet state is set to “01” and beta is “01”, which represents 75% channel goodness. The power threshold is set to “00000” so number of transmitted packets is set to 3 at 3600 ns (1 clock delay). When the power threshold is set to “00010”, the number of transmitted packets is set to 1 at 3800 ns (0 clock delay). When the value of beta is changed, the output takes 2 clock cycles to change as shown in 4200 ns. The difference in delay can be attributed to the nature of the FSM.

## 9. Conclusions

In this paper, the aim was to assess the relationship between delay and power for a wireless transmission system. For the given system, two scenarios were investigated, channel-aware model and BACA model. We proposed an iterative enhanced algorithm for BACA system to solve such a problem. The results indicate that by using the algorithm, we achieve the delay power tradeoff suboptimal curve with less complexity than other methods. Additionally, in the case of the channel-aware model, this research extends our knowledge of the influence of channel conditions and arrivals distribution on performance metrics. Interpolation was used to correlate the relation between performance metrics, average arrival rate, and channel goodness factor. This analysis provides an estimation for the performance metrics given different parameters.

In the case of the BACA model, we examined the impact of channel goodness factor and the distribution of the arrival packets on the power-delay tradeoff curve. This provides a deeper insight into the parameters that affect the performance metrics.

The results of this research support the idea that the tradeoff curve is piecewise linear, convex and decreasing. Each point represents a different policy in one threshold. While the findings of this investigation complement those of earlier studies, they are very important for wireless technology and IoT applications. They provide a deeper insight into the limitations of the system to get a reasonable range of performance metrics.

A hardware implementation based on FPGA for a simplified channel aware scheduler is introduced. The design occupies less than 1% of the available resources. The proposed design shows that the maximum power consumption is 98.5 mW and the maximum processing clock speed is 190 MHz. As a future work, the authors would like to fully implement a BACA policy and further investigate the power delay tradeoff.

## Figures and Tables

**Table 1:** System Parameters.

Symbol	Definition	Value
a[n]	The number of arriving data packets at the end of n^th^ time slot	a∈{0,1,…,A}
s[n]	The number of served data packets in timeslot n	s∈{0,1,…,S}
q[n]	Queue length at the beginning of time slot n	q∈{0,1,…,Q}
h[n]	Channel state in time slot n	w∈{good, bad}
$\alpha_{a}$	The probability of having a arriving packet	$\alpha_{a}\geq 0$
β	The probability of having good channel state	0<β≤1
$\bar{a}$	Average arrival rate	
$P_{s}^{w}$	The power of transmitting s packets in w channel state	
$f_{q,s}^{w}$	The probability of transmitting s packets under good channel state with queue length is equal to q	
F	The total transmission policy matrix	
$\tau_{i,j}$	Probability of transition from state i to state j	
$\mu_{i,j}$	Probability of transition from state i to state j with j<i	
$\lambda_{i,j}$	Probability of transition from state i to state j with j≥i	
π	The queue states probability distribution under steady-state	
D	System Delay	
P	Estimated Power Consumption	

**Table 2:** System Power Consumption Calculations.

	Power under Good Channel State	Power under Bad Channel State
s=0	$P_{0}^{good}=0\;J$	$P_{0}^{bad}=0\;J$
s=1	$P_{1}^{good}=9.0\times 10^{-14}\;\;J$	$P_{1}^{bad}=36\times 10^{-14}\;\;J$
s=2	$P_{2}^{good}=18.2\times 10^{-14}\;J$	$P_{2}^{bad}=72.8\times 10^{-14}\;J$
s=3	$P_{3}^{good}=59.5\times 10^{-14}\;J$	$P_{3}^{bad}=238\times 10^{-14}\;J$

**Table 3:** Place and Route Results.

	Available	Proposed Scheduler
**Total Logic Elements**	114,480	143
**Total Registers**		41
**Total Memory Bits**	3,981,312	0
**Embedded Multiplier 9-bit Elements**	532	0
**Power Consumption (mW)**		98.5
**Max. Freq. (MHz)**		190
